# Consequences of undertreatment of hypothyroidism

**DOI:** 10.1007/s12020-023-03460-1

**Published:** 2023-08-09

**Authors:** Ulla Feldt-Rasmussen, Grigoris Effraimidis, Sofie Bliddal, Marianne Klose

**Affiliations:** 1 Department of Medical Endocrinology and Metabolism, Rigshospitalet, Copenhagen University Hospital, Copenhagen, Denmark; 2 Institute of Clinical Medicine, Faculty of Health and Clinical Sciences, Copenhagen University, Copenhagen, Denmark; 3 Department of Endocrinology and Metabolic Diseases, Larissa University Hospital, Faculty of Medicine, School of Health Sciences, University of Thessaly, Larissa, Greece

**Keywords:** Thyroid function, Age, Levothyroxine, Treatment, Optimisation

## Abstract

**Purpose:**

To provide an overview of consequences of undertreatment with levothyroxine (LT4) in the common non-communicable disease, hypothyroidism.

**Methods:**

Narrative review of the literature.

**Results:**

Hypothyroidism is globally very prevalent at all age groups and represents a non-communicable disease in which the risks and consequences are preventable. In children and adolescents, the most devastating consequences of undertreatment are poor growth and development. Lack of early treatment in congenital hypothyroidism can lead to permanent damage of brain function. In young to middle-aged adults, consequences are often overlooked, and treatment delayed by many years. The resulting consequences are also at this age group compromised brain and physical functioning but less severe and partly reversible with treatment. The undertreated condition often results in a higher risk of several secondary devastating diseases such as increased cardiovascular disease burden, obesity, hypertension, poor physical capacity, poor quality of life. In young women of fertile age the consequences of undertreatment with LT4 are subnormal fertility, recurrent pregnancy loss, preeclampsia, compromised fetal growth and neurocognitive development. There is a further risk of 30–50% of developing postpartum thyroiditis. In the elderly population care must be given to avoid confusing a slightly high serum TSH as result of physiological age adaptation with a requirement for LT4 treatment in a truly hypothyroid patient.

**Conclusion:**

Undertreatment of the preventable non-communicable disease hypothyroidism requires more focus both from caretakers in the healthcare system, but also from the global political systems in order to prevent the personally devastating and socioeconomically challenging consequences.

## Introduction

Hypothyroidism (or myxoedema) is globally a very common endocrine disorder depending on the composition of the various populations’ age, sex, race, genetics, environmental factors such as iodine and selenium intake as well as the specific diagnostic criteria and many other influences [1]. It belongs among the non-communicable diseases. In typical cases of overt hypothyroidism, the diagnosis is not difficult, but there are several grey zones such as how to manage the milder or subclinical cases, and the differences in reference ranges across the age groups.

Myxoedema was first treated successfully in 1891 when George Redmayne Murray diagnosed a 46-year-old woman with the disease. He prescribed an extract from sheep thyroid. The patient improved significantly within a few weeks. Murray published this first account of a human patient with hypothyroidism given substitution with thyroid extract (injected subcutaneously) [2]. Clinically, the effect was beyond doubt and the patient lived another 28 years on thyroid substitution—eventually to die of cardiac failure in 1919 [3]. Soon after the use of thyroid extract ingested by mouth was verified as being efficient in the treatment as well [4], Desiccated thyroid was much used for many years and as late as 1978 leading British endocrinologists felt compelled to warn against its use [5]. It was still being marketed for many years and has recently had a revival in its use as ‘natural thyroid substitution’ [1].

Thyroxine was not chemically identified until 1927 [6]. About 25 years later, Gross and Pitt-Rivers [7] as well as French investigators detected the second thyroid hormone—triiodothyronine (T3) [8, 9]. Hart and Maclagan in 1950 [10] reviewed the use of thyroxine (and particularly L-thyroxine (LT4)), which had been available since the 1930s but had not gained wide acceptance (maybe because of its initial high cost)—despite its obvious advantages. For the past many decades, LT4 has been commercially available and the cornerstone in treatment of hypothyroidism, despite several challenges mainly by patients and patient organisations to shift to combination therapy with T3 [1] or to revert to desiccated thyroid preparations [1], none of which is recommended in current guidelines.

The aim of this narrative review is not to discuss above alternative choices, but to give evidence for the long-term efficacy of LT4 and the risks and consequences of undertreatment throughout different ages and patient populations.

## Causes of hypothyroidism

Globally, the main cause for spontaneous hypothyroidism is still overt iodine deficiency or partial iodine deficiency for special conditions such as pregnancy. A large population study in Denmark [11] reported that the most common subtype (present in 84.4% of patients) was spontaneous (presumably autoimmune) hypothyroidism, followed by postpartum (4.7%) and amiodarone-induced hypothyroidism (4.0%). Less common causes were subacute thyroiditis (1.8%), previous radiation or surgery (1.8%) to the thyroid gland, congenital hypothyroidism (1.6%) and lithium-associated (1.6%) thyroid failure. Nowadays, iatrogenic causes have become more frequent due to various immunotherapies [11]. Rarer causes include central (secondary)- or primary congenital hypothyroidism, thyroid hormone resistance, overtreatment with antithyroid drugs, or effects of other drugs, ingestion of food containing goitrogens (e.g. vegan food), therapeutic or environmental irradiation [1].

## Physiology of thyroid function and diagnosis of hypothyroidism

**Fig. 1:**
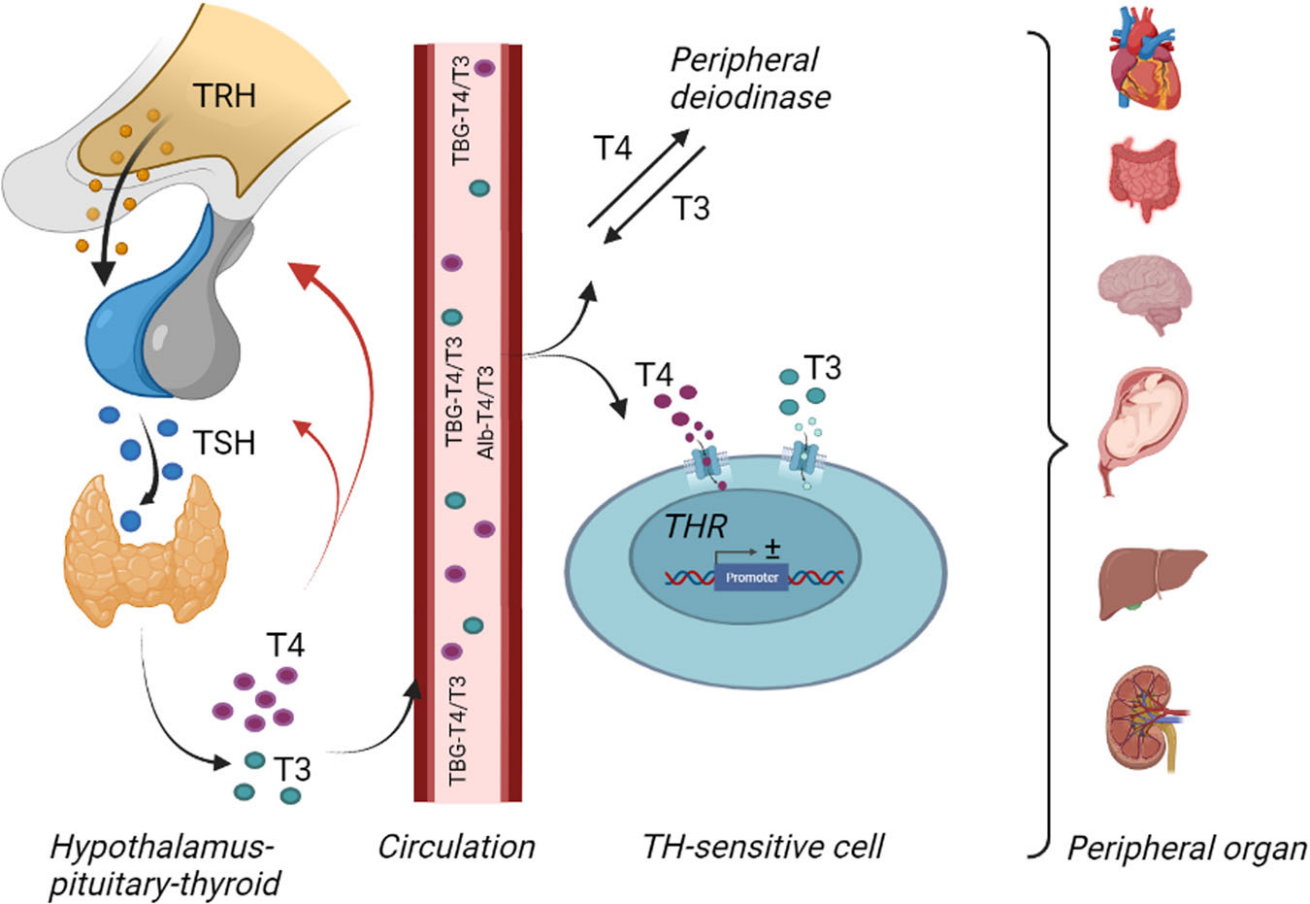
Overview of thyroid hormone actions. Thyroid hormone production and secretion are regulated by a negative feedback mechanism with the hypothalamus producing thyrotropin releasing hormone in response to low concentrations of T3 and T4, which again stimulate the anterior pituitary to produce and secrete TSH, resulting in stimulation of the thyroid gland to increase T3 and T4 production. The far majority of thyroid hormones are circulated in the blood stream bound to proteins until reaching target organs. Alb albumin, T3 triiodothyronine, T4 thyroxine, TBG thyroid binding globulin, TH thyroid hormone, THR thyroid hormone receptor, TRH thyrotropin releasing hormone, TSH thyroid stimulating hormone. Created with BioRender.com

The diagnosis of overt hypothyroidism can sometimes be done entirely on the phenotypical features of the disease, but in most cases biochemical measurement of thyroid function variables is necessary, particularly in subtle cases. In some situations, it will also be needed to measure supportive biomarkers [12]. Thyroid hormone secretion is regulated in a negative feedback manner by pituitary thyrotropin (TSH), which in turn is stimulated by the TSH releasing hormone (TRH) (Fig. 1). Obtaining a normal euthyroid state is thus a delicate balance between the hypothalamus-pituitary function and that of the thyroid gland, the two of which are regulated in a logarithmic (TSH)/linear (T4 and T3) way. The diagnosis of primary hypothyroidism is based on biochemical demonstration of low circulating T4 in the face of an elevated serum TSH, which is in typical and overt cases usually not difficult (see later).

The reliability of the biochemical measurements on the one hand, and the cut off values of the various hormone measurements between normal and hypothyroid persons on the other, have therefore become crucial for a correct diagnosis. Due to above-mentioned log/linear relationship, serum TSH is the most sensitive variable to assess a change in thyroid function, which in the case of diagnosing primary hypofunction means an increase beyond the upper reference limit of normal people. A crude choice for a universally accepted reference range has been 0.4–4.0 mU/l, but this has turned out to be too simplistic, since there are huge variations among populations in terms of environmental influence such as iodine intake, age, genetics and many others. Serum TSH is thus higher in areas of both overt and partial iodine deficiency [13], very high in neonates [14], variable during pregnancy according to gestation [15], increasing at high age [16] and higher in some population groups such as Ashkenazy Jews [17]. Circulating thyroid hormones show similar reference range differences although less well explored [16]. Consequently, current reference ranges could potentially lead to inappropriate commencement of treatment in older individuals and in certain populations with other genetic traits than those normally included in the international guidelines. On the other hand, it could result in undertreatment of younger and middle-aged groups. There is not yet evidence to support the general use of age-appropriate reference intervals, nor to understand the impact of thyroid hormone variations in younger individuals.

## Undertreatment in children and adolescents

Severe congenital hypothyroidism will untreated result in cretinism with blunted growth, very low IQ, generally failed neurological development and psychological disturbances. In the Western part of the world, such cases should be completely irradicated when a neonatal TSH screening programme was introduced, taking advantage of the high concentration just after birth allowing for use of dried blood spots. However, despite this available technology since the early 1970s, ~70% of the World’s population does not have access to it, and therefore children still develop cretinism [18]. The diagnosis of congenital hypothyroidism usually cannot be done on purely clinical grounds until the child is several months and often much later. The consequent treatment delay therefore leaves these children with permanent neurological damage. To increase the possibility of capturing also milder cases of congenital hypothyroidism, the TSH level for further work up of thyroid function has been lowered, and free T4 measurements have been added to capture also congenital central hypothyroidism [19, 20]. Also, for these neonatal measurements local reference intervals are important for correct interpretation [20].

The causes of hypothyroidism occurring later in childhood and in adolescents include Iodine deficiency, subtle congenital defects in e.g. thyroid hormone synthesis, hereditary thyroid disorders (thyroid hormone resistance), autoimmune thyroid disease, and potentially environmental endocrine disruptors. Such late occurrence is usually associated with milder symptoms, but also resulting in impaired growth, behavioural problems and symptoms of Attention-Deficit/Hyperactivity Disorder (ADHD), conditions which might be reversible if treated early and correctly with LT4. The most prominent consequences of not treating or undertreating hypothyroidism in children are related to a failure of general and neuropsychological development with poor ability to perform in school and later, failure of obtaining a proper education and thus they very often fail to become independent adults. This preventable disorder, therefore, also has high socioeconomic consequences for the individual and society.

## Undertreatment in adults after puberty until old age

After attaining final height and pubertal development young/middle-aged adults are also at risk of underdiagnosis and -treatment for hypothyroidism mostly due to very non-specific symptomatology and few typical features [1]. Since thyroid hormones are responsible for cellular metabolism in all mammalian organs, it is not surprising that symptoms can occur from all organ systems if thyroid hormones are reduced even only slightly. The risks of undertreatment of a diagnosed hypothyroid state are related to poor mental function, ADHD or other behavioural conditions, reduced cardiac output and decreased physical capacity due to the low cardiac output combined with muscle weakness and reduced lung function. A reflex overstimulation of catecholamines leads to hypertension, which together with the tendency to obesity subsequently leads to an increased cardiovascular disease burden. The patients often have hair loss, dry skin and constipation and due to a slow metabolism patients have a low production of many substances e.g. haemoglobin, adrenal glucocorticoids; conversely there is a low degradation of many substances giving rise to e.g. elevated liver enzymes and they have a low glomerular filtration rate. Doses of many drugs may have to be reduced due to the slow metabolism and thereby risk of accumulation or direct poisoning. All of these symptoms and signs result in a poor quality of life and poor general work-functioning [1].

An important risk factor for undertreatment is suboptimal thyroxine treatment owed to gastrointestinal comorbidities. Alterations of gastric pH will affect the absorption of levothyroxine such as seen in patients with helicobacter pylori infection, chronic atrophic gastritis, gastroparesis, or in simultaneous treatment with drugs interfering with gastric acidic output; conditions that taken together will affect up between 20–50% of the world’s population during their life span[21, 22]. Furthermore, chronic autoimmune gastritis (resulting in pernicious anaemia) and coeliac disease are among the most common other autoimmune diseases overlapping with thyroid autoimmunity, although expectedly vastly underdiagnosed[23, 24]. The risk of polyautoimmunity is also important to take into account in patients with consistent complaints of low quality of life despite achieved euthyroidism [25, 26].

Other factors may also increase the risk e.g. protein loss in proteinuria, which should automatically lead to increase of the LT4 replacement due to urinary loss of LT4, and several other drugs or food components can give rise to interference with the gastrointestinal absorption of the LT4.

## Undertreatment in women during reproductive age

Several special circumstances are related to treatment of hypothyroidism in women of reproductive age. First, patients using oral contraceptives with oestrogen will have an increase in the total concentrations of circulating binding proteins, such as thyroid hormone binding globulin (TBG) and albumin, and thus total T4 will be elevated. This will disturb measurements of free T4 because most available immunoassays are unable to properly correct for the extremes found in the high end of the correction curves. Thus, free T4 measurements cannot stand alone in interpretation of treatment effect in young women on oral contraception. Second, the same challenge occurs in pregnancy where the high oestrogen concentrations result in similar risks of misinterpreted thyroid function test results. However, in pregnancy, a correct dosage is not only important to the woman but also to secure proper fetal development and to reduce the risk of pregnancy complications such as pregnancy loss and preterm birth [27, 28]. Physiologically, the maternal thyroid hormone production adapts to this situation upon the binding of the pregnancy-related hormone human chorionic gonadotropin to the TSH receptor resulting in the necessary increased thyroid hormone production [29]. In women on LT4 replacement therapy the treatment will need to be adjusted to mimic the physiological response securing transfer of sufficient LT4 from the maternal blood stream to the fetus [12, 30].

Third, women with hypothyroidism will often experience irregular menstrual periods and infertility, and some will even be diagnosed with hypothyroidism as a result of the internationally recommended screening for thyroid dysfunction of all women referred to fertility treatment [30, 31]. A particular challenge is the treatment of women who will undergo controlled ovarian stimulation in which a steep increase in oestrogen concentration acutely stimulates the liver to increase the production of TBG, and thus, the need for increased thyroid hormone production as well [32]. A recent meta-analysis by Busnelli et al. demonstrated that in women treated for hypothyroidism, TSH concentrations increase ~1.5 mIU/l upon controlled ovarian stimulation and remains increased in more than 3 months in up to 50% of the women [32]. To account for this, women who are treated with LT4 for hypothyroidism will need a higher dose than in pre-pregnancy/pre-fertility treatment to avoid undertreatment during the critical phase of implantation and early pregnancy. Even women presenting with subclinical hypothyroidism may benefit from LT4 substitution prior to fertility treatment and pregnancy as demonstrated in large observational studies [33, 34]. However, slight aberrations in thyroid function may not need to be treated given the risk of overtreatment leading to (iatrogenic) preeclampsia and preterm birth [34], and in light of the lack of evidence of a benefit on offspring IQ in randomised controlled trials [35, 36].

Fourth, women with autoimmune hypothyroidism have a risk of undertreatment with LT4 in the postpartum period, where they may develop postpartum thyroiditis with an initial destructive thyroiditis with release of thyroid hormone (if any left in the thyroid gland) and hyperthyroidism, always followed by a hypothyroid phase until regeneration of the thyroid function (if any left). The hypothyroid phase most often requires an increased dose of LT4, which may eventually become a permanent and lifelong requirement, but may in some cases be transitory [37]. The most prominent consequences for these women are to have limited capacity to take care of their child due to fatigue, while at the same time get a diagnosis of postpartum depression. All women with unexpected symptoms postpartum should have their thyroid function tested in order to discover and treat hypothyroidism.

## Undertreatment of the elderly and people with cardiac diseases

A variety of factors complicate not only the diagnosis and management of hypothyroidism in the elderly but also the determination of the hypothyroid elderly who receive inadequate thyroid hormone therapy.

TSH reference ranges in the elderly must be evaluated in the light of the age-dependent shift in serum TSH distribution towards higher concentrations with increasing age. Studies in several populations (Americans, Scotish, Ashkenazi Jews, Chinese) showed that TSH concentrations progressively increased with age and the 97.5th percentiles of the reference population are considerably higher in the elderly (older than 70 years old) than in younger adults [38–41]. This shift cannot be explained only by the higher prevalence of thyroid autoimmunity in the older population [42, 43]. It might reflect an age-related alteration in the TSH set point and/or reduced TSH bioactivity and/or reduced sensitivity of the thyroid gland to TSH, since longitudinal data suggest an inter-individual age-dependent TSH increment not associated with a decline in free T4 [42].

The association between the age-related changes in thyroid function and longer life expectancy has been investigated in both animal and humans. An association between increased longevity and reduced thyroid function was found in small and large mammals [44–46]. In humans, population-based studies have observed an association between increased serum TSH levels and/or lower levels of thyroid hormones and extended life span. Whether the association between the decline in thyroid hormone activity and longevity is a positive adaptation or a progressive, negative deterioration, is still not clear. It has been proposed that the lower basal metabolic rate and the subsequent slow catabolism due to the lower free T4 activity can possibly explain this association [47]. However other studies failed to support this hypothesis [48].

The numerous medications that older people usually take due to increased (multi)morbidity can affect the thyroid function tests not only by interfering with the synthesis, transport, and metabolism of TSH and thyroid hormones but also by interfering with thyroid function immunoassays. A recent large cohort study found that ~1/3 of older hypothyroid patients on LT4 treatment had taken at least one medication that interfere with thyroid hormones [49]. In addition, it is known that common chronic conditions such as heart, kidney, liver disease, diabetes, major depression, as well as low caloric intake, which are more prevalent in the elderly, may result in changes in thyroid function as part of the euthyroid sick syndrome. A detailed careful review of the drugs and the comorbidities should always be conducted before an older individual with hypothyroidism treated with LT4 is classified as under-treated [42].

The age-dependent shift in serum TSH distribution towards higher concentrations with increasing age and the observed relation between longevity and the lower thyroid function prompted experts to recommend a higher TSH level target when older individuals with overt or subclinical hypothyroidism are treated [50]. Consequently, a mildly elevated TSH in older hypothyroid patients receiving LT4 treatment is acceptable (e.g. TSH of 6 miU/l in a 70-year-old patient), after other factors (drugs, comorbidities) affecting the thyroid function tests have been taken into consideration. 

However, several studies showed that not sufficiently treated overt and/or subclinical hypothyroidism is associated with increased early mortality risk in older patients [51–54]. In addition, a recent population-based, retrospective cohort study found that the risk of cardiovascular mortality increased progressively with higher TSH levels, as compared with euthyroid individuals, especially in the older adults [55]. It must be mentioned that these studies used different criteria and different number of TSH measurements regarding the classification of undertreated patients, but in any case, these findings highlight the importance for further studies on the optimal TSH target in older hypothyroid patients.

The profound negative effect of hypothyroidism on cardiac performance, particularly in patients with pre-existing heart failure, and the beneficial effect of LT4 therapy on cardiac function should always be taken into consideration when evaluating the adequacy of the thyroid hormone therapy [42, 56, 57]. A recent retrospective cohort study of more than 150,000 patients with incident hypothyroidism of whom 97% received LT4 therapy during the follow-up period, showed that the association between the highest TSH concentration and increased risk of heart failure was present in both patients younger and older than 65 years, whereas the association with increased risk of ischaemic heart disease remained significant only in patients aged 65 years or under [53]. On the contrary, the risk of stroke/transient ischaemic attack was not increased in all patients. These findings combined with the above-mentioned association between cardiovascular mortality and higher TSH [55], point towards an intense LT4 treatment in the hypothyroid patient.

## Undertreatment with levothyroxine in other special situations

Some special patient situations are inherently combined with more challenging management of hypothyroidism replacement, and therefore require special attention in order to avoid undertreatment with LT4. Although there are not many peer-reviewed publications in relation to these rare special situations, they are worth mentioning, because they are clinically relevant and important for proper management of patients with hypothyroidism. Based on lack of scientific evidence, the statements below are mainly expert opinion based.

In hypothalamus-pituitary-hypothyroidism or central/secondary myxoedema, the thyroid hypofunction is due to a failing pituitary production of biologically active TSH. Unfortunately, the biologically inactive TSH molecule is measurable by the TSH immunoassays and therefore useless in the diagnosis of hypothyroidism as well as monitoring of replacement [58]. Total and free T4 measurements (free T4 estimates) are thus the only useful tools by current methodology [59]. In case of multiple pituitary hormone deficiencies, the situation becomes more complex in women of fertile age on oestrogen replacement therapy (or on oral contraceptive drugs). This is the same situation concerning evaluation as mentioned above for pregnancy, except worse, since TSH cannot be used at all [60]. These females are therefore at high risk of under-replacement with consequences of a worse metabolic profile [61].

Patients in intensive care units (ICU) are also particularly problematic because they often cannot take tablets orally. In these situations, patients need other administrations either i.v LT4 or oral liquid formulation if they have a gastric tube [62]. In the monitoring of the biochemical thyroid function it is important to realise that all patients in ICU have non-thyroidal illness (NTI) [63]. This implies a peripheral reduced conversion of T4 (in replaced patients of the LT4 replacement dose) to T3 and an increase to reverse (inactive) T3, which is not routinely measured. There may therefore be both central and peripheral changes not related to the replacement itself, so the best is to continue the regular replacement dose independent of the false thyroid function measurements in order to avoid under-replacement [64]. When the NTI is alleviated, the regular replacement can be adjusted if needed.

After a thyroidectomy, a variety of dosing issues of LT4 can occur, depending first of all on the underlying thyroid disease i.e. cancer, thyroid autoimmunity or non-toxic goitre. Some patients are quite easy to adjust to a correct dose of LT4, but others may be more challenging e.g. the case of thyroid cancer surgery, where patients may often need inappropriately high LT4 doses to keep TSH low. Although there is a high risk of severe overdosing in the initial phase of the cancer, achieving the correct maintenance dose can take time considering the long half-life of T4 and can subsequently often result in a longer period of under-replacement. Quality of life seems to depend more on degree of hypothyroidism than the level of thyroid surgery (total or partial thyroidectomy) [65], and it therefore makes sense to finetune the dose in small increments and explain to the patient why the process cannot be speeded up. Thus, by optimising LT4 monotherapy it may perhaps be possible to reduce the number of complaints.

## Conclusions

Hypothyroidism is globally very prevalent at all age groups and represents a non-communicable disease in which the risks and consequences are preventable. However, the diagnosis is often delayed for long periods with resulting consequences for the individual and the society. Hypothyroidism as a non-communicable disease is not part of any governmental focus. Undertreatment of the preventable non-communicable disease hypothyroidism therefore requires more focus both from caretakers in the healthcare system, but also from the global political systems in order to prevent the personally devastating and socioeconomically challenging consequences.
